# Two new species of *Pseudohadena* Alphéraky, 1889 from Kazakhstan (Lepidoptera, Noctuidae, Xyleninae)

**DOI:** 10.3897/zookeys.187.2661

**Published:** 2012-04-27

**Authors:** Oleg Pekarsky

**Affiliations:** 1 H-1068, Budapest, Felsőerdősor u. 16-18, Hungary

**Keywords:** Lepidoptera, Noctuidae, *Pseudohadena*, *Jaxartia*, new species, Ustyurt, Kazakhstan

## Abstract

Two new species of *Pseudohadena*, *Pseudohadena anatine* **sp. n.** and *Pseudohadena gorbunovi* **sp. n.** (Lepidoptera, Noctuidae) are described from Ustyurt, Kazakhstan. Illustrations of adults and the genitalia of both sexes are provided. Microstructures of vesicae are described and illustrated. A diagnostic comparison is made with *Pseudohadena evanida psammoxantha* Ronkay, Varga & Fábián, 1995, *Pseudohadena magnitudinis* Hacker & Ebert, 2002 and *Pseudohadena pseudamoena* (Boursin, 1943).

## Introduction

*Pseudohadena* Alphéraky, 1889 is a Central Asian genus of the subfamily Xyleninae. It was re-described by Ronkay et al. in 1995 and divided into 3 subgenera, *Pseudohadena* Alphéraky, 1889, *Jaxartia* Püngeler, 1914 and *Pseudopseustis* Hampson, 1910.

*Jaxartia* was established at the beginning of last century by Püngeler in 1914 for the species *Jaxartia elinguis*, described by him in the same article. The second taxon in the genus, *Jaxartia striolata*, was discovered and described by Filipjev in 1949. At the end of the 20^th^ century, the genus *Pseudohadena* was revised by Ronkay et al. (1995) and *Jaxartia* was downgraded to a subgenus of *Pseudohadena*, comprising a further four species (*Pseudohadena jordana* Staudinger, 1900, *Pseudohadena evanida* Püngeler, 1914, *Pseudohadena pseudamoena* Boursin, 1943 and *Pseudohadena cymatodes* Boursin, 1954).

In 2007, Fibiger and Hacker listed 9 species belonging to the subgenus *Jaxartia* in Noctuidae Europaeae volume 9 and arranged them into 3 species-groups. The last known species was described in 2008 by Shirvani et al., thereby the subgenus comprises 10 species.

During a study of the Noctuidae material collected by Pavel Gorbunov on the plateau Ustyurt, Western Kazakhstan in 2010, two formerly unknown *Pseudohadena* species were recognised. Both new species are externally similar to *Pseudohadena (Jaxartia) evanida* and six other representatives of the subgenus have clearly recognisable differences in their external and genital features.

## Systematic part

### Pseudohadena (Jaxartia) anatine

Pekarsky sp. n.

urn:lsid:zoobank.org:act:031F6A87-1F52-4771-8328-3E451F6A58D2

http://species-id.net/wiki/Pseudohadena_anatine

Figs 1–4

#### Holotype.

Male, S.W. KAZAKHSTAN, Ustyurt Res., Kendyrli (spring), 128 m, 42°57'N, 54°41'E, 3.x.2010, leg. P. Gorbunov; slide No.: OP1055m (coll. O. Pekarsky, deposited in the HNHM Budapest).

#### Paratypes. 

4 males, with same data as holotype, slide No: OP1155m (male) (coll. O. Pekarsky); 1 male, with same data as holotype (coll. P. Gorbunov); 2 males , S.-W. KAZAKHSTAN, Ustyurt Nature Reserve, Kendyrli (spring), 128 m, 42°57'N, 54°41'E, 11.10.09 (coll. P. Gorbunov); 1 male & 1 female S.-W. KAZAKHSTAN, Ustyurt Nature Reserve, Oneri (cordon & spring), 42°36'N, 54°08'E, 12.10.09 (coll. P. Gorbunov); 2 males , S.-W. KAZAKHSTAN, Ustyurt Nature Reserve, Kendyrli (spring), 128 m, 42°57'N, 54°41'E, 28.09.10 (coll. P. Gorbunov); 1 male, Kazakhstan, Mangistauskaya, Karakiyanskyi, Ustyurt Plat., 9–15.10.2009, leg. V. Zurilina (coll. M. Dvořák), 1 female (coll. O. pekarsky); 2 males, SW Kazakhstan, Mangistau prov., Karakiya distr., Sand Tynyshtyk, 43°06'53"N, 054°11'39"E, 5.x.2010, leg. V. Zurilina (coll. O. Pekarsky); 1 female SW Kazakhstan, Ustyurt Res., Karynzharyk Sands, Saksorka, 42°42'N, 54°06'E, 7.v.2010 leg. P. Gorbunov, slide No: OP1193f (female) (coll. O. Pekarsky).

#### Diagnosis.

*Pseudohadena anatine* is placed in the subgenus *Jaxartia* due to its widely bipectinate male antenna and the digging armature of the foreleg consisting of 4 large, curved spine-like setae outside on the basitarsus and an additional spinose seta inside at its distal end (Figs 53, 54). In addition, the vestiture of the head and the thorax is bifurcated hair-like scales (Fig. 52), which is a conspicuous apomorphic character of the subgenus. It is a member of the *evanida* species-group, having a rather broad, less lanceolate fore wing with an indistinct noctuid pattern, strong valvae with subapical dilatation, large costal plate and semiglobular-ovoid corpus bursae.

The new species resembles *Pseudohadena (Jaxartia) evanida evanida*, *Pseudohadena (Jaxartia) evanida psammoxantha*, *Pseudohadena (Jaxartia) pseudamoena* and *Pseudohadena (Jaxartia) deserticola* by the beige-grey coloration of the fore wing with indistinct wing pattern; *Pseudohadena (Jaxartia) magnitudinis* and *Pseudohadena (Jaxartia) cymatodes* differ from *Pseudohadena (Jaxartia) anatine* by their well-developed characteristic wing pattern; *Pseudohadena (Jaxartia) leucochlora* can be distinguished from all mentioned congeners by its characteristic greenish fore wing ground colour. The broadly bipectinate male antenna of the new species is similar to those of *Pseudohadena magnitudinis*, *Pseudohadena cymatodes* and *Pseudohadena evanida*. *Pseudohadena (Jaxartia) anatine* can be distinguished externally from the closely related *Pseudohadena (Jaxartia) evanida* and *Pseudohadena (Jaxartia) magnitudinis* by the shorter and broader fore wing with an almost straight costal margin and less acutely pointed apex. The wing pattern of the new species is regularly more obsolescent than in the two closest relatives; however, rather unicolorous, less distinctly-marked specimens can be found in both *Pseudohadena (Jaxartia) evanida* and *Pseudohadena (Jaxartia) magnitudinis*. The male genitalia of the new species differ from those of the above-mentioned two species in the shape of the clasper, the size and shape of the digitus, and the structure of the vesica. Also, all related species have clearly recognisable differences in the microstructure of the walls of vesica. The new species is distinguishable from its closest relatives, *Pseudohadena evanida* and *Pseudohadena magnitudinis*, by its fairly curved, medially thicker clasper, small, spiculiform distal process of the digitus, and small, conical distal diverticulum of the vesica, whereas *Pseudohadena evanida* and *Pseudohadena magnitudinis* have a shorter, evenly wide, ribbon-like, less curved clasper, a much larger tooth-like distal process of the digitus, and a larger, much longer distal diverticulum of the vesica. The distal diverticulum of *Pseudohadena anatine* is covered by push-pin-like spinules with broad bases (Fig. 24), whereas the surface of the distal diverticulum is armed with elongated spinules with narrow bases in related species (Figs 35, 39).

The female genitalia of the new species differ from those of its relatives by the size of the entire organ, the shape and length of the ductus bursae, the shape of the corpus bursae and the shape of subgenital plate (8^th^ abdominal segment). *Pseudohadena anatine* is easily separable from its closest relatives by the smaller size of the genitalia (total length 8 mm), the shorter and wider ductus bursae, and the acute edges of the subgenital plate. *Pseudohadena evanida* has larger genitalia (total length 8.5–9 mm), longer and narrower ductus bursae, a narrower subgenital plate with quadrangular edges. *Pseudohadena magnitudinis* is characterized by the even larger size of the female genitalia (total length 10 mm), longer ductus bursae and rather quadrangular edges of subgenital plate. In addition, the apophyses of the new species are as long as the ductus bursae, whereas the apophyses of related species are shorter than the ductus bursae.

#### Description.

**Figures 1–8.**
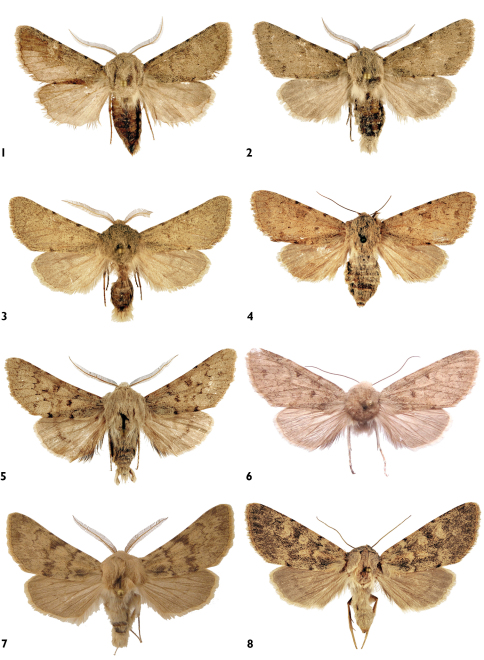
Adults. **1–4** *Pseudohadena (Jaxartia) anatine* sp. n. **1** holotype male, Kazakhstan **2** paratype male, Kazakhstan **3** paratype male, Kazakhstan **4** paratype female, Kazakhstan **5–6** *Pseudohadena (Jaxartia) evanida psammoxantha* **5** paratype male, Kazakhstan **6** holotype female, Kazakhstan. **7–8** *Pseudohadena (Jaxartia) magnitudinis* **7** male, Iran **8** female, Iran.

Male (Figs 1–3). Wingspan 39–40 mm, length of fore wing 17 mm. Head, thorax, abdomen and fore wing beige grey; latter irrorated by black scales. Thorax and head covered with bifurcated hair-like scales (Fig. 52) some of which have black tip. Usual hair-like scales on metathorax long, thin, directed across abdomen. Black hair-like scales around eyes long and dense. Palpi short, covered with black hair-like scales on outer side and light-beige scales on inner side. Forewing broad and short with obtuse apex; costa straight; outer margin has almost straight termen. Wing pattern very indistinct, basal and subbasal lines marked as costal spots only; antemedial line represented by a few diffuse darker spots; medial line consists of a dark costal patch and shadow-like fascia; postmedial crossline traceable by black scales on veins; subterminal line variably strong, sinuous, marked by smaller or larger brown-grey arrowheads. Orbicular and reniform stigmata also less discernible, relatively large, roundish with light margins; claviform stigma diffuse or obsolete. Terminal line fine, continuous, grey brown; cilia long, narrow, variably strongly striated with dark brown. Hindwing pale, shining beige grey, transverse line present, discal spot pale but usually recognisable. Female (Fig. 4). As male but remarkably larger in size (wingspan 47 mm), with more obtuse apex of forewing.

**Figures 9–20.**
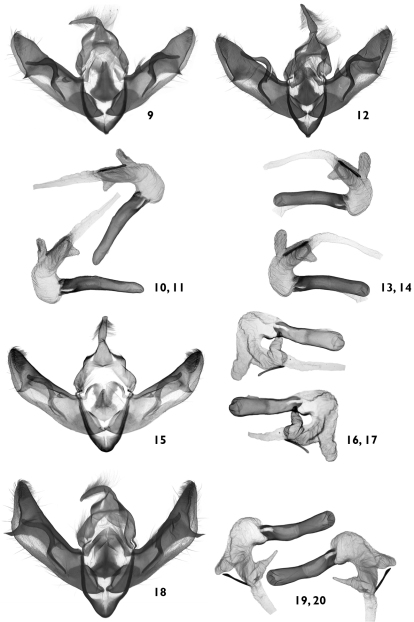
Male genitalia. **9–11** *Pseudohadena (Jaxartia) anatine* sp. n., male genitalia, holotype, Kazakhstan, slide No. OP1055m **9** clasping apparatus **10** aedeagus (ventral view) **11** aedeagus (ventral view, opposite side). **12–14** *Pseudohadena (Jaxartia) anatine* sp. n., male genitalia, paratype, Kazakhstan, slide No. OP1155m **12** clasping apparatus **13** aedeagus (ventral view) **14** aedeagus (ventral view, opposite side). **15–17** *Pseudohadena (Jaxartia) evanida psammoxantha*, male genitalia, paratype, Kazakhstan, slide No. RL4982m **15** clasping apparatus **16** aedeagus (ventral view) **17** aedeagus (ventral view, opposite side). **18–20** *Pseudohadena (Jaxartia) magnitudinis*, male genitalia, Iran, slide No. OP1071m **18** clasping apparatus **19** aedeagus (ventral view) **20** aedeagus (ventral view opposite side).

**Figure 21.**
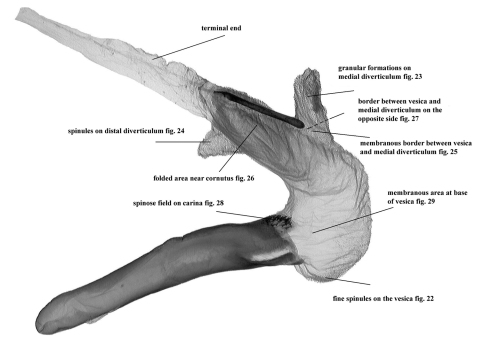
Vesica characteristics of *Pseudohadena (Jaxartia) anatine* sp. n., Kazakhstan.

**Figures 22–29.**
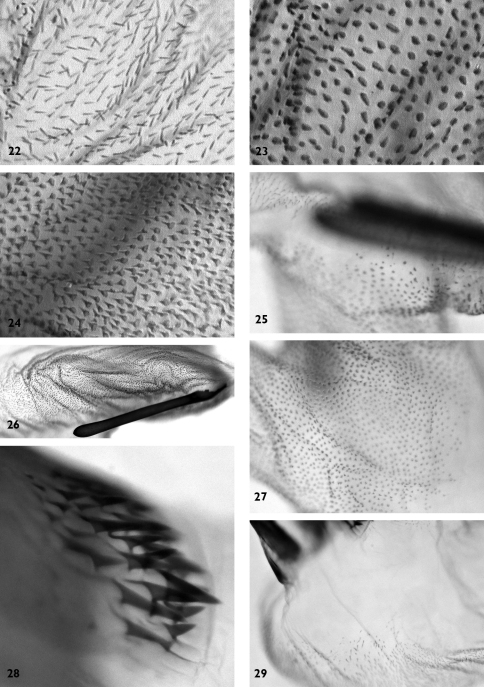
Vesica microstructure of *Pseudohadena (Jaxartia) anatine* sp. n. **22** fine spinules on the vesica **23** granular formations on medial diverticulum **24** spinules on distal diverticulum **25** membranous border between vesica and medial diverticulum **26** folded area near cornutus **27** border between vesica and medial diverticulum on opposite side **28** spinose field on carina **29** membranous area at base of vesica.

**Figures 46–51.**
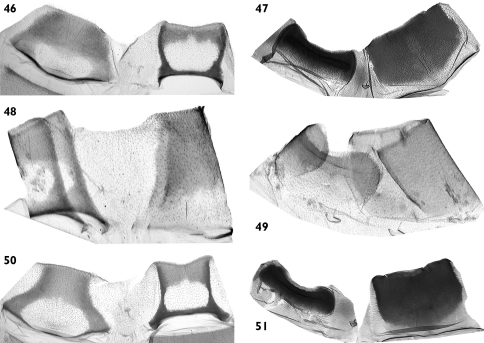
Abdominal segments. **46** *Pseudohadena (Jaxartia) anatine* sp. n., Kazakhstan, 8^th^ abdominal segment of the male **47** 7^th^ abdominal segment of female **48** *Pseudohadena (Jaxartia) evanida psammoxantha*, 8^th^ abdominal segment of male **49** 7^th^ abdominal segment of female **50** *Pseudohadena (Jaxartia) magnitudinis*, 8^th^ abdominal segment of the male **51** 7^th^ abdominal segment of the female.

**Figures 52–60.**
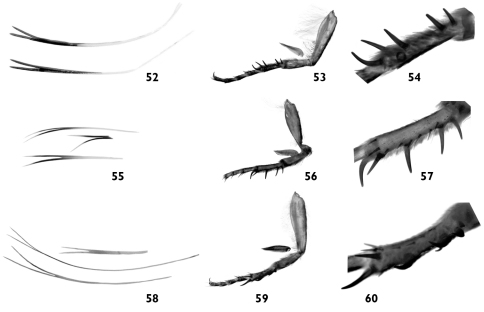
**52** Bifurcated hair-like scales from head and thorax of *Pseudohadena (Jaxartia) anatine* sp. n., Kazakhstan **53** right fore leg with separated epiphysis **54** basitarsus of fore leg **55** bifurcated hair-like scales from head and thorax of *Pseudohadena (Jaxartia) evanida psammoxantha* **56** right fore leg with separated epiphysis **57** basitarsus of fore leg **58** bifurcated hair-like scales from head and thorax of *Pseudohadena (Jaxartia) magnitudinis* **59** right fore leg with separated epiphysis **60** basitarsus of fore leg.

**Male genitalia** (Figs 9–14, 21–29). Genital armature well sclerotized; uncus strong, hairy, with flattened and pointed tip; tegumen slender, moderately wide, 1.4 times shorter than vinculum; penicular lobes narrow, bearing long setae; juxta subdeltoidal with wide basal (ventral) plate and long triangular dorsal extension; vinculum sclerotized, rather V-shaped. Valvae symmetrical, massive, with widely parallel margins; cucullus triangular with pointed apex, corona weak; sacculus small with rounded, dorsal margin setose (clavus reduced); costa almost straight with small subapical salience; editum conspicuous, setose; clasper long, medially curved, thicker in middle and thinner at end, resembling head of a duck; costal plate large, digitus subapical, surpassing ventral margin of valva, small and spiculiform, very wide at base; ventral margin of valva and central area between sacculus and clasper weakly sclerotized. Aedeagus cylindrical, curved ventrally in distal part; carina with spinose field (Fig. 28). Vesica tubular, wider basally, everted forward, recurved laterally, scobinate with fine spinules (Fig. 22) except an area at base of proximal part (Fig. 29); partly membranous in proximal and medial parts; terminal end without scobination; medial diverticulum ratherlarge, scobinate, scobinations granular (Fig. 23); membranous clear space at border between spinules on main corpus of vesica and granules on medial diverticulum (Fig. 25), whereas opposite side of vesica and medial diverticulum with same granulose character of scobination (Fig. 27); distal diverticulum conical with broad base and narrower upper part, with scobination consisting of push-pin-like spinules with broad bases (Fig. 24). Terminal cornutus straight, long, strong and narrow, with rounded tip; folded area near cornutus covered by fine spinules (Fig. 26). Eight abdominal segment with characteristic sclerotized structures on both sides (Fig. 46); tergite with two symmetrical, well-sclerotized curved bars, connected by a cross-section bar in the anterior and weakly-sclerotized band in posterior part; posterior margin slightly concave; middle section of the tergite unsclerotized and looks like a rectangular window with rounded lateral margins, straight anterior margin and convex posterior margin; sternite tapering with sclerotized curved anterior and straight posterior margins with unsclerotized narrow ”window” anteriorly.

**Figures 61–63.**
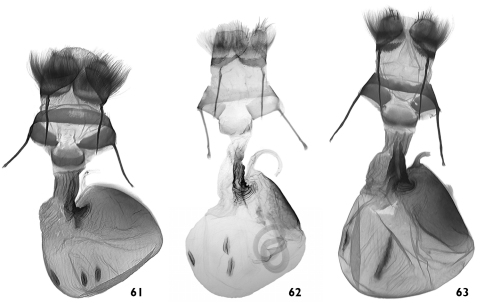
Female genitalia. **61** *Pseudohadena (Jaxartia) anatine* sp. n., female genitalia, paratype, Kazakhstan, slide No. OP1193f **62** *Pseudohadena (Jaxartia) evanida psammoxantha*, female genitalia, holotype, Kazakhstan, slide No. RL5182 **63** *Pseudohadena (Jaxartia) magnitudinis*, female, Iran slide No. OP1096f.

**Female genitalia** (Fig. 61). Ovipositor short, broad, papillae anales densely hairy, setae on apical edges short, sparse. Apophyses anteriores slender, apophyses posteriores thin with acute tips, as long as ductus bursae. Ostium bursae broad, short, ventral plate narrow. Ductus bursae short, broad, membranous with coarse wrinkles, its lateral sclerotization extending to appendix bursae and continuing ventrad. Appendix bursae small, rounded, well sclerotized, area near ductus bursae wrinkled and with large lateral sclerotized plate. Corpus bursae small, beveled ovoid, with three broad, short signa. Seventh abdominal segment heavily sclerotized; tergite a fully sclerotized pentagonal plate with parallel lateral sides and straight posterior margin; sternite narrow, strongly sclerotized with diffuse, almost straight anterior and concave posterior margins (Fig. 47).

#### Note.

The study of a large number of *Pseudohadena* moths belonging to different subgenera and species groups showed a lot of variability of some parts in their genital structure. For instance, the terminal cornutus is sometimes doubled (Figs 13, 14), occurring rarely in larger series of moths with ordinary structure.

#### Etymology.

The species name refers to the duck-like shape of the clasper.

#### Distribution.

The species is known only from its type-locality, South-west Kazakhstan, Ustyurt plateau.

### Pseudohadena (Jaxartia) gorbunovi

Pekarsky sp. n.

urn:lsid:zoobank.org:act:420F7C70-8868-46C8-9141-44991C6A6FD9

http://species-id.net/wiki/Pseudohadena_gorbunovi

Figs 64–67

#### Holotype.

Male, SW KAZAKHSTAN, Ustyurt Res., 4 km S of Kokesem cordon, 316 m, 43°08'N, 54°54'E, 1–2.x.2010, leg. P. Gorbunov; slide No.: OP0976m (coll. O. Pekarsky, deposited in the HNHM Budapest).

#### Paratypes.

23 males, with same data as holotype (coll. O. Pekarsky); 3 males, 2 females, SW KAZAKHSTAN, Ustyurt Plat., 30km S Sai-Utes, Syndy, 223 m, 44°00'N, 53°25'E 19.ix.2010 leg. P. Gorbunov, (coll. O. Pekarsky); 4 males, 1 female, SW Kazakhstan, Mangistau Prov., Ustyurt Nat. Res., Cordon Kokesem, 43°10'10"N, 54°53'16"E, 1.10.2010 leg. V. Zurilina (coll. O. Pekarsky); 56 males, 3 females, from same locality, 2.x.2010, leg. V. Zurilina (coll. O. Pekarsky); 1 male, Kazakhstan, Mangistau Prov., Cordon Kokesem, Ustyurt nat. reserv., 12.10.2010, (coll. M. Dvořák), 1 male, Kazakhstan, Mangistauskaya, Karakiyanskyi, Ustyurt Plat., 9–15.10.2009, leg. V. Zurilina (coll. M. Dvořák), 6 males, SW Kazakhstan, Mangistau prov., Ustyurt Nat. Res., Cordon Kenderly, 42°57'28"N, 54°41'34"E, 3.x.2010, leg. V. Zurilina (coll. O. Pekarsky); 11 males, SW KAZAKHSTAN, Ustyurt Nat. Res., Kendyrli (spring), 128 m, 42°57'N, 54°41'E, 29.ix.2010, leg. P. Gorbunov, (coll. O. Pekarsky); 29 males, from same locality, 3.x.2010, slide Nos: OP1056m, OP1057m (males), 2 females, 10.x.2010, leg. P. Gorbunov, slide Nos: OP1153f, OP1154f (female) (coll. O. Pekarsky); 20 males, SW Kazakhstan, Mangistau prov., Karakiya distr., Sand Tynyshtyk, 43°06'53"N, 54°11'39"E, 5.x.2010, leg. V. Zurilina (coll. O. Pekarsky); 6 males, from same locality, 4.x.2010, V. Zurilina (coll. O. Pekarsky); 7 males, 1 female, SW KAZAKHSTAN, Ustyurt Plateau, 19 km N of Beineu, 45°30'N, 55°15'E, 120 m 8.x.2010, leg. P. Gorbunov, slide Nos: OP1059m, OP1060m (males), OP0977f (female) (coll. O. Pekarsky); 10 males, SW KAZAKHSTAN, Ustyurt Res., Tynyshtyk Boget at Karashek Mt., 43°06N, 54°11E 4.x.2010, leg. P. Gorbunov, slide No.: OP1058m (male) (coll. O. Pekarsky); 1 male, SW KAZAKHSTAN, Beket-Ata 20 km S, 268 m, 43°29'35.2"N, 54°01'37.5"E, 7.10.2010, leg. K. Nupponen, slide No.: OP1067m (male) (coll. O. Pekarsky); 1 female, Kazakhstan, Mangistauskaya, Karakiyanskyi, Ustyurt Plat., 9-15.10.09, leg. V. Zurilina, (coll. O. Pekarsky); 4 males, SW Kazakhstan, Mangistau Prov., Ustyurt Nat. Res., Cordon Kokesem, 43°10'10"N, 54°53'16"E, 2.10.2010, leg. V. Zurilina (coll. O. Pekarsky, deposited in ZISP, St. Petersburg).

#### Diagnosis.

*Pseudohadena (Jaxartia) gorbunovi* possesses all diagnostic external characters of the subgenus *Jaxartia* (wide bipectinate antenna of males, five curved spine-like setae on basitarsus of fore leg, bifurcated hair-like scales on thorax and head). It resembles *Pseudohadena evanida*, *Pseudohadena leucochlora* and *Pseudohadena pseudamoena*. The genitalia structure of both sexes, especially the coiled type of vesica, the long ductus bursae, and the size of corpus bursae and appendix bursae indicates the close relationship with *Pseudohadena cymatodes* and *Pseudohadena pseudamoena*. Despite of the conspicuous differences in the habitus of *Pseudohadena gorbunovi* and *Pseudohadena cymatodes*, the latter species is the closest relative of *Pseudohadena gorbunovi*.

The external features of *Pseudohadena gorbunoviare* compared below with those of *Pseudohadena pseudamoena* due to the striking external differences between it and *Pseudohadena cymatodes*. The main distinguishing external features of *Pseudohadena gorbunoviare* smaller size, the almost straight antemedial line, the more elongated fore wing with acute apex and oblique outer margin, and the narrower hind wing; *Pseudohadena pseudamoena* has a zigzagged antemedial line, much wider fore wings with less oblique outer margin and obtuse apex and the hind wing is also wider, more rounded. The two species also differ in the structure of bifurcated hair-like scales (Figs 101, 104).

The diagnostic features of the male genitalia are in the shape of the cucullus, the costal process (digitus), the clasper, and the structure of vesica; those of the female genitalia are the shapes of the corpus bursae and appendix bursae. The new species differs from related species by its elongated, uniform clasper, fine, attenuated distal process of the digitus, relatively short cucullus, and the dorso-ventral direction of the twist of the vesica. In comparison with *Pseudohadena gorbunovi*, *Pseudohadena pseudamoena* has a larger, medially dilated, “butter knife”-shaped clasper with acute tip, somewhat shorter, wider distal process of the digitus, a much longer cucullus, and the vesica is twisted in a ventro-dorsal direction.

The female genitalia of *Pseudohadena gorbunovi* are distinguishable from those of *Pseudohadena pseudamoena* by the almost equally sized and similarly shaped corpus bursae and appendix bursae, whhereas in *Pseudohadena pseudamoena* the corpus bursae is smaller than the appendix bursae.

The detailed characterisation of the genitalia of *Pseudohadena cymatodes* will be presented in a separate paper (Pekarsky, in prep.).

#### Description.

**Figures 64–69.**
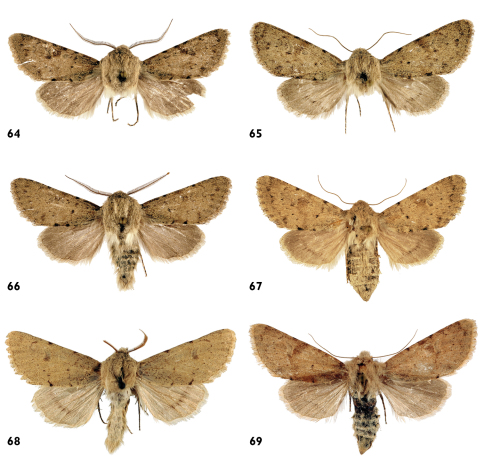
Adults. **64–67** *Pseudohadena (Jaxartia) gorbunovi* sp. n. **64** holotype male, Kazakhstan **65** paratype female **66** paratype male **67** paratype female. **68–69** *Pseudohadena (Jaxartia) pseudamoena*. **68**paratype male, Armenia **69** female, Iran.

Male (Figs 64, 66). Wingspan 31–40 mm, length of fore wing 15–17mm. Head, thorax, abdomen and fore wing beige; fore wing irrorated with a few blackish-brown scales, thorax and head mixed sparsely with black-tipped hair-like scales. Scales on thorax and head bifurcated except on metathorax, which is covered by unforked hair-like scales directed across abdomen. Eye surrounded by black hair-like scales. Palpus short, wide, densely covered by long black hair-like scales on outer side and light-beige scales on inner side. Forewing narrow, with acute apex; costa straight; outer margin oblique. Wing pattern indistinct: basal, subbasal and medial lines recognisable only on most strongly patterned specimens; antemedial line represented by some darker spots; medial line most often represented only by dark costal patch; postmedial line curved and dentate; subterminal line curved, composed by blackish-brown scales; orbicular stigma with darker patch in centre; reniform stigma narrow, lunulate; claviform stigma diffuse, with small darker basal patch; terminal line present, cilia striated with dark brown. Hind wing pale, shining beige grey; transverse line present; discal spot hardly discernible.

Female (Figs 65, 67). Wingspan 36 mm, length of fore wing 15–17 mm. External characters as for male but with more rounded fore wing; wings and abdomen somewhat darker in colouration.

**Figures 70–75.**
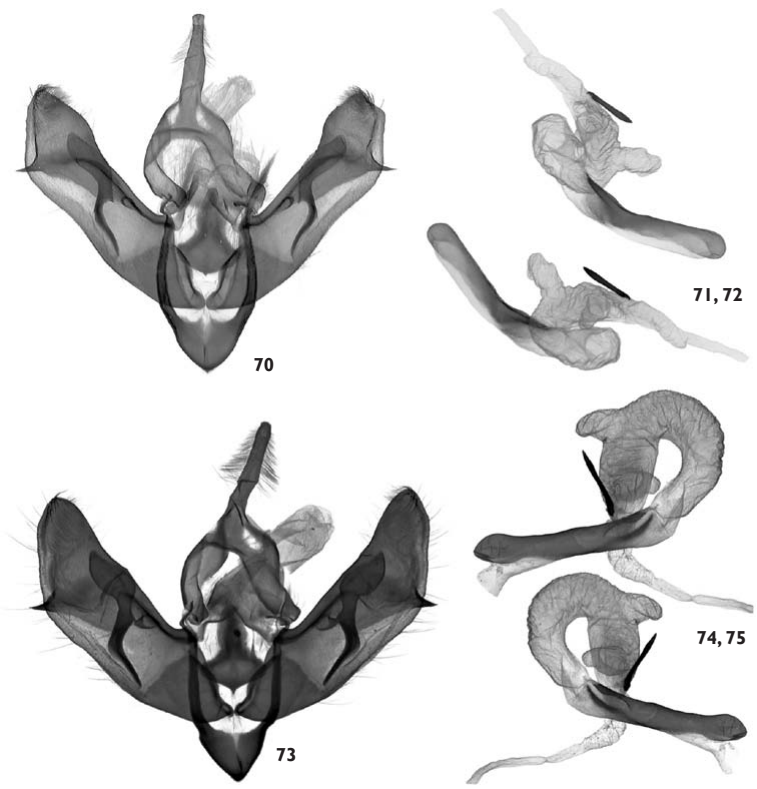
Male genitalia. **70–72** *Pseudohadena (Jaxartia) gorbunovi* sp. n., male genitalia, holotype, Kazakhstan, slide No. OP0976m **70** clasping apparatus **71** aedeagus (ventral view) **72** aedeagus (ventral view, opposite side) 73–75 *Pseudohadena (Jaxartia) pseudamoena*, male genitalia, paratype, Armenia, slide No. OP1198m **73** clasping apparatus **74** aedeagus (ventral view) **75** aedeagus (ventral view, opposite side).

**Figure 76.**
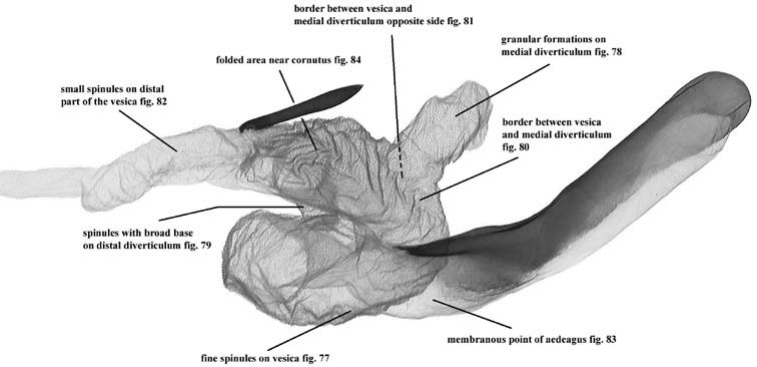
Vesica characteristics of *Pseudohadena (Jaxartia) gorbunovi* sp. n., holotype, Kazakhstan, Slide No. OP0976m.

**Figures 77–84.**
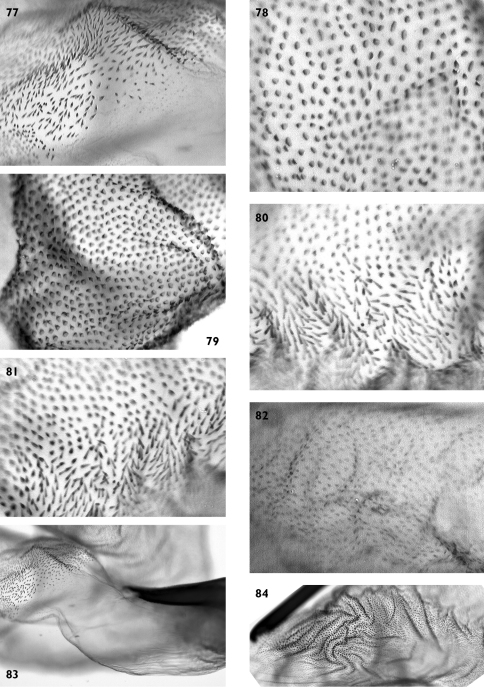
Vesica microstructure of *Pseudohadena (Jaxartia) gorbunovi* sp. n. **77** fine spinules on vesica **78 **granular formations on medial diverticulum **79** spinules with broad bases on distal diverticulum **80** border between vesica and medial diverticulum **81** border between vesica and medial diverticulum on opposite side **82** small spinules on distal part of vesica **83** membranous point of aedeagus **84** folded area near cornutus.

**Figures 97–100.**
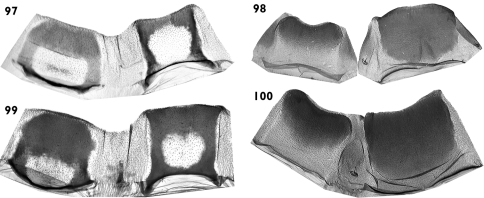
Abdominal segments. **97** *Pseudohadena (Jaxartia) gorbunovi* sp. n., 8^th^ abdominal segment of male **98** 7^th^ abdominal segment of female **99** *Pseudohadena (Jaxartia) pseudamoena*, 8^th^ abdominal segment of male **100** 7^th^ abdominal segment of female.

**Figures 101–106. 101:**
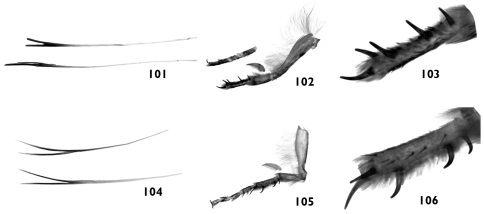
Bifurcated hair-like scales from head and thorax of *Pseudohadena (Jaxartia) gorbunovi* sp. n., Kazakhstan **102** right fore leg **103** basitarsus of fore leg **104** bifurcated hair-like scales from head and thorax of *Pseudohadena (Jaxartia) pseudamoena*, Iran **105** right fore leg **106** basitarsus of fore leg.

**Male genitalia** (Figs 70–72, 76–84). Genital armature well sclerotized; uncus strong, flattened with obtuse flattened dorsal-ventral apex; tegumen ribbon-like, 0.67 times length of vinculum; penicular lobes small, bearing long setae; juxta shield-like with rounded basal (ventral) side and elongated (dorsal) extension; vinculum V-shaped. Valvae symmetrical, massive, wide, with parallel sides; sacculus short, triangular with dorsal setose sector; costa straight from base to cucullus, with big salience subapically; editum granule-shaped, setose; clasper strong, wide, flattened, medially curved; central area of the valva between sacculus and costal process weakly sclerotized; costal process large, its extension thin, acute, spiculiform, situated subapically; corona weak. Aedeagus cylindrical, distal part curved ventrally; ventral part sclerotized; carina heavily sclerotized, without spines. Vesica tubular, everted forward and recurved ventrally producing a full coil and continued in opposite direction from carina in a subconical tube; basal tube and medial part thick walled; medial third with long subconical diverticulum; distal third with somewhat shorter diverticulum and with long, robust, pointed terminal cornutus; basal part of vesica with membranous area with clear surface (Fig. 83); lateral and dorsal surfaces of vesica covered by fine spinules from basal area towards medial diverticulum (Fig. 77); medial diverticulum covered with granule-like formations with acute tips (Fig. 78); vesica with a strict border between two different types of scobination on main tube of vesica (spinules) and medial diverticulum (granulous formations with acute tips) on both sides (Figs 80, 81); scobination of distal diverticulum consists of spinules with broad bases (Fig. 79); terminal end of vesica covered by small spinules (Fig. 82). Eighth abdominal segment with characteristic sclerotized structures on both sides (Figs 97, 98); tergite with two symmetrical, well-sclerotized, anteriorly curved bars, connected by a cross-bar anteriorly and by weakly sclerotized band posteriorly; posterior margin slightly concave; middle section of tergite has no sclerotization and looks like a rectangular window with rounded lateral margins and straight posterior and anterior margins; sternite rather oval with wide sclerotization posteriorly and straight anterior and posterior margins, with unsclerotized "window" anteriorly.

**Figures 107–110.**
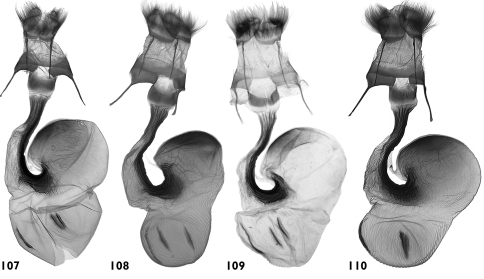
Female genitalia. **107** *Pseudohadena (Jaxartia) gorbunovi* sp. n., female genitalia, paratype, Kazakhstan, slide No. OP0977f **108** *Pseudohadena (Jaxartia) gorbunovi* sp. n., female genitalia, paratype, Kazakhstan, slide No. OP1163f **109** *Pseudohadena (Jaxartia) pseudamoena*, female genitalia, Iran, Elburs, slide No. RL8103f **110** *Pseudohadena (Jaxartia) pseudamoena*, female, Iran, slide No. OP1126f.

**Female genitalia** (Figs 107, 108).

Ovipositor short; papillae anales densely hairy. Apophyses anteriores thin with small spatulate tips; apophyses posteriores somewhat longer than apophyses anteriores. Ostium bursae broad, ventral plate sclerotized, quadrangular with rounded lower corners, its walls scobinate. Ductus bursae long, tubular, sinusoid, a sclerotized crest running laterally from ostium bursae to apical part of corpus bursae. Appendix bursae as large as corpus bursae, most parts sclerotized; corpus bursae elliptical-semiglobular with three unequal signa: first signum long, tapering towards its tip; second signum medium-long, ribbon-like, equally wide throughout; third signum similar to second one but somewhat shorter. Seventh abdominal segment heavily sclerotized; tergite a fully sclerotized plate with parallel lateral sides and convex posterior and anterior sides; sternite smaller having a narrow, strongly sclerotized posterior part with rounded posterolateral corners, concave centrally; anterior part lightly sclerotized (Fig. 98).

#### Etymology.

The new species is dedicated to the famous Russian entomologist, Mr. Pavel Gorbunov, who collected both of the new species of *Pseudohadena* described herein.

#### Distribution.

The species is known only from its type-locality, South-west Kazakhstan, Ustyurt plateau.

## Supplementary Material

XML Treatment for Pseudohadena (Jaxartia) anatine

XML Treatment for Pseudohadena (Jaxartia) gorbunovi

## Figures and Tables

**Figure 30.**
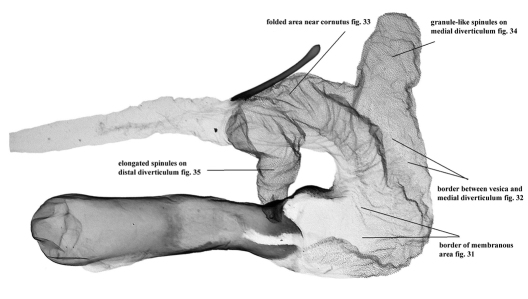
Vesica characteristics of *Pseudohadena (Jaxartia) evanida psammoxantha*, paratype, Kazakhstan, slide No. RL4982m

**Figures 31–35.**
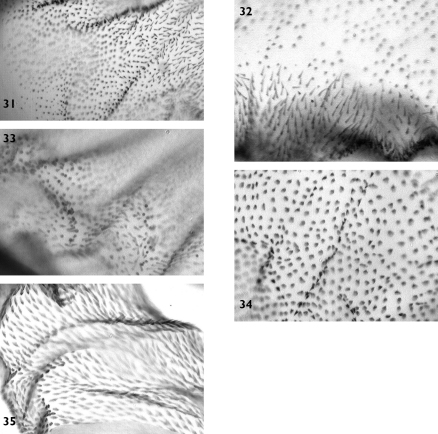
Vesica microstructure of *Pseudohadena (Jaxartia) evanida psammoxantha*, paratype, Kazakhstan, slide No. RL4982m **31** border of membranous area **32** border between vesica and medial diverticulum **33** folded area near cornutus **34** granule-like spinules on medial diverticulum **35** elongated spinules on distal diverticulum.

**Figure 36.**
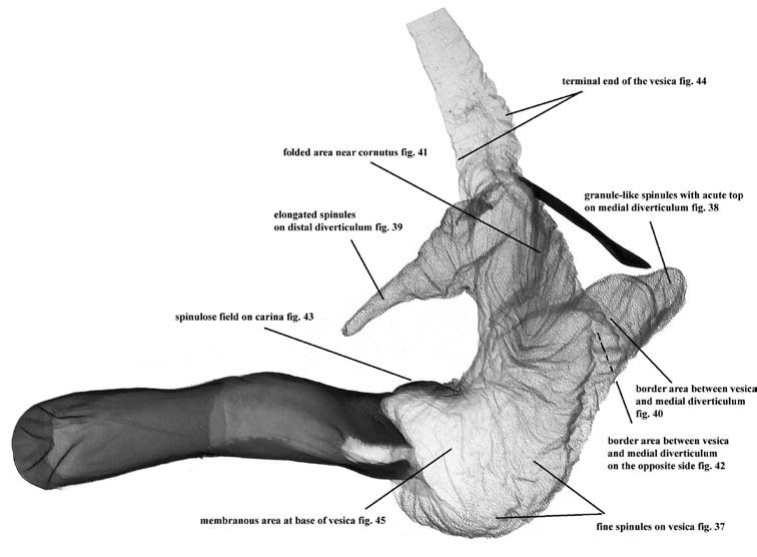
Vesica characteristics of *Pseudohadena (Jaxartia) magnitudinis*, Iran, slide No. OP1071m

**Figure 37–45.**
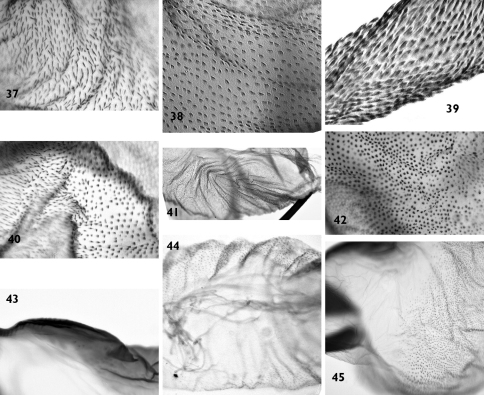
Vesica microstructure of *Pseudohadena (Jaxartia) magnitudinis*, Iran, slide No. OP1071m **37** fine spinules on vesica **38** granule-like spinules with acute tips on medial diverticulum **39** elongated spinules on distal diverticulum **40** border between vesica and medial diverticulum **41** folded area near cornutus **42** border area between vesica and medial diverticulum on opposite side **43** spinulose field on carina **44** terminal end of the vesica 45 membranous area at base of vesica.

**Figure 85.**
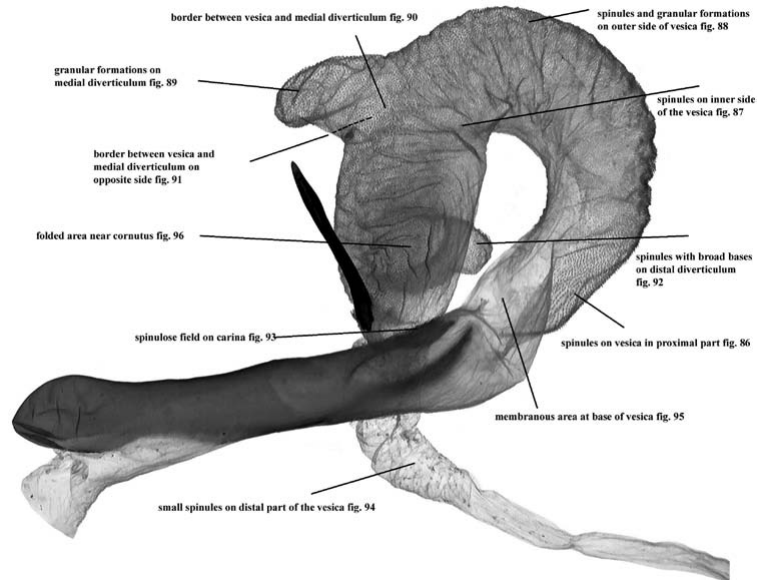
Vesica characteristics of *Pseudohadena (Jaxartia) pseudamoena*, paratype, Armenia, slide No. OP1198m

**Figures 86–96.**
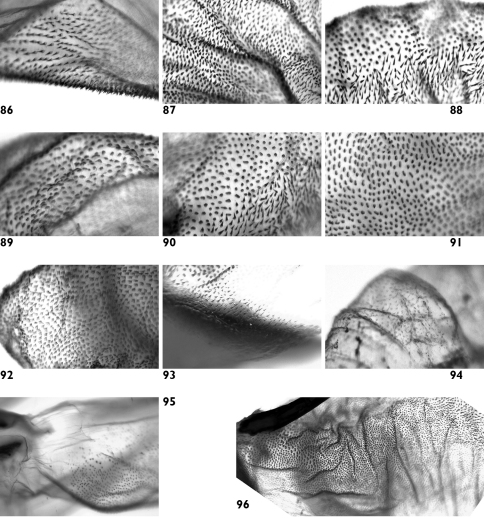
Vesica microstructure of *Pseudohadena (Jaxartia) pseudamoena*. **86** spinules on vesica in proximal part **87** spinules on inner side of vesica **88** spinules and granular formations on outer side of vesica **89 **granular formations on medial diverticulum **90** border between vesica and medial diverticulum **91 **border between vesica and medial diverticulum on opposite side **92** spinules with broad bases on distal diverticulum **93** spinulose field on carina **94** small spinules on distal part of vesica **95** membranous area at base of vesica **96** folded area near cornutus.
